# Serum vitamin D levels are decreased in patients without remission of Graves’ disease

**DOI:** 10.1007/s12020-012-9789-6

**Published:** 2012-09-15

**Authors:** Tetsuyuki Yasuda, Yasuyuki Okamoto, Noboru Hamada, Kazuyuki Miyashita, Mitsuyoshi Takahara, Fumie Sakamoto, Takeshi Miyatsuka, Tetsuhiro Kitamura, Naoto Katakami, Dan Kawamori, Michio Otsuki, Taka-aki Matsuoka, Hideaki Kaneto, Iichiro Shimomura

**Affiliations:** 1 Department of Metabolic Medicine, Osaka University Graduate School of Medicine, 2-2 Yamadaoka, Suita, Osaka, 565-0871 Japan; 2 Sumire Clinic, Osaka, Japan

## Introduction

Graves’ disease (GD) is an autoimmune thyroid disease in which thyrotropin receptor autoantibodies (TRAb) cause hyperthyroidism. Although medical treatment with antithyroid drugs (ATD) is the first choice treatment for GD in Japan and Europe, a remission rate of GD with ATD is not satisfactory, and many patients need long-term treatment with ATD or further treatments such as radioactive iodine therapy or thyroidectomy [1]. Therefore, it is very important to identify the factors relating to the remission of GD. It has been recently shown that vitamin D deficiency is associated with the onset and/or development of several autoimmune diseases, including multiple sclerosis (MS), inflammatory bowel disease (IBD), and type 1 diabetes (T1DM) [2]. Furthermore, it has been reported more recently that patients with autoimmune thyroid diseases including GD have lower vitamin D status [3, 4]. However, there is no study comparing vitamin D status between the patients with and without remission of GD. In the present study, we examined vitamin D status in female patients with and without remission of GD and discussed the role of vitamin D in the pathogenesis and/or prognosis of GD.

## Patients and methods

We recruited consecutive female GD patients who could achieve and not achieve remission by the treatment of ATD from the subjects who attended the Sumire clinic for following up GD in summer and autumn periods in 2011. Patients with renal disease, hepatic disease and malignancy, and taking medications that affect vitamin D status were excluded from the study. GD was diagnosed by clinical and biochemical symptoms of hyperthyroidism, the presence of diffused goiter and TRAb positivity. Patients with remission of GD (remission group, *n* = 18) were defined as those who could maintain a euthyroid status for more than 1 year after the discontinuation of ATD. Patients without remission of GD (non-remission group, *n* = 36) were defined as those who could not discontinue ATD for more than 4 years after the initiation of therapy and were still positive for TRAb. Forty-nine control healthy female subjects who had normal thyroid function without antithyroid peroxidase antibody (TPOAb) and antithyrogloblin antibody (TgAb) were recruited in the same periods. This study was approved by the ethical committee of Sumire clinic, and informed consent was obtained from all patients. Casual blood samples were obtained in summer and autumn periods in 2011. Vitamin D status was evaluated by the measurement of serum 25(OH)D_3_ levels using the competitive protein binding assay (Mitsubishi Chemical Medience, Tokyo, Japan). An analytical sensitivity, a mean minimum detectable level, and intra- and inter-assay coefficients of variation for 25(OH)D_3_ were 2.5–320, 4.1 ng/ml, and less than 10.9 %, respectively. Serum free T4 (FT4) levels were measured by competitive enzyme immunoassay (Tosoh, Tokyo, Japan). Serum TSH, TRAb, TPOAb, and TgAb levels were measured by two-site immunoenzymometric assay (Tosoh, Tokyo, Japan). Statistical analyses were performed using Stat-View statistical software (Version. 5.0 for Windows; Abacus Concepts, Berkeley, CA). Continuous variables are expressed as mean ± SD. Differences between patients with GD and control subjects were examined by the Mann–Whitney *U* test or unpaired Student’s *t* tests as appropriate. Associations of serum 25(OH)D_3_ levels with variables were examined by the Spearman’s rank correlation coefficient. A two-sided value of *P* < 0.05 was considered statistically significant.

## Results

Clinical characteristics in patients with and without remission of GD and control subjects are shown in Table 1. Durations from the discontinuation of ATD in remission group were 2.0 ± 1.1 years. Durations from the initiation of ATD were significantly longer in non-remission group than in remission group (9.2 ± 4.8 vs 6.1 ± 2.5 years, *P* < 0.05). As shown in Table 1, serum 25(OH)D_3_ levels were significantly lower in non-remission group than in remission and control group (14.5 ± 2.9 vs 18.2 ± 5.1 ng/ml, *P* < 0.005, and 18.6 ± 5.3 ng/ml, *P* < 0.0005). On the other hand, there was no significant association between serum 25(OH)D_3_ levels and serum TRAb levels in non-remission group.

**Table 1:** Clinical characteristics

	Control	Graves’ disease	
		Remission	Non-remission
Number of subjects	49	18	36
Age (years)	37.3 ± 6.9	38.7 ± 5.5	37.8 ± 8.1
TSH (μU/ml)	1.92 ± 0.87	1.40 ± 0.94	1.45 ± 1.60
Free T4 (ng/dl)	1.11 ± 0.17	1.09 ± 0.10	1.19 ± 0.44
TRAb positivity (%)	n.d.	16.7	100^#^
Current dose of ATD (mg/day)	None	None	9.0 ± 8.0^a^
Treatment time (years)	None	6.1 ± 2.5	9.2 ± 4.8^##^
25(OH)D3 (ng/ml)	18.6 ± 5.3	18.2 ± 5.1	14.5 ± 2.9*^,^**

Date are means ± standard deviations

^#^ *P* < 0.0001 (vs remission); ^##^ *P* < 0.05 (vs remission); * *P* < 0.005 (vs remission); ** *P* < 0.0005 (vs control)

^a^Dose are expressed by the comparable dose of methimazole (50 mg of propylthiouracil is converted to 5 mg of methimazole)

## Discussion

In the present study, we demonstrated that serum vitamin D levels were significantly lower in female GD patients without remission than in those with remission. To our best knowledge, this is the first report showing the significant difference of vitamin D status between the patients with and without remission of GD. In the present study, we did not evaluate the differences of the severity of hyperthyroidism in onset of GD and duration of hyperthyroidism between the two groups. In addition, durations from the initiation of ATD were significantly longer in non-remission group than in remission group. However, it has been reported that vitamin D status is not related with the severity of hyperthyroidism, and is not changed by the treatment for GD [4, 5]. Therefore, it is unlikely that the significant differences of vitamin D status between the patients with and without remission of GD are attributed to the differences of the severity and duration of hyperthyroidism and duration of the treatment for GD.

Vitamin D is known for its primary role in bone and mineral homeostasis, and its deficiency is associated with cardiovascular disease, cancer, and adiposity as well as osteoporosis [6]. Interestingly, it has been shown recently that vitamin D has potent immunomodulatory effects and plays important roles in the pathogenesis of autoimmune diseases. Vitamin D inhibits the production of Th1 polarizing cytokine (IL-12), thereby, indirectly shifting the polarization of T cells from a Th1 toward a Th2 phenotype. In the CD4^+^ T cell response, vitamin D directly inhibits the production of Th1 cytokines (IL-2 and IFN-γ), and enhances Th2 cytokine (IL-4) production [2]. In addition, recent numerous studies have shown the relation of vitamin D and various autoimmune diseases. Vitamin D receptor (VDR) gene polymorphisms and vitamin D status are associated with different autoimmune diseases such as MS, IBD, and T1DM [2]. Furthermore, vitamin D supplementation prevented the onset and/or development of several kinds of autoimmune diseases in humans and animal models [2]. These results suggest that vitamin D deficiency likely causes the onset and/or development of several kinds of autoimmune diseases.

GD is an autoimmune thyroid disease in which TRAb causes hyperthyroidism. The pathogenesis of GD is complicated and involved in multiple genetic and environmental factors. Recent studies have demonstrated a role of vitamin D in GD. Serum vitamin D levels are decreased and associated with thyroid volume in female patients with new onset GD [4]. Vitamin D-related gene polymorphisms such as VDR gene and vitamin D binding protein gene are associated with GD [7, 8]. Vitamin D deficiency modulates Graves’ hyperthyroidism induced by thyrotropin receptor immunization in BALB/c mice [9]. It is well known that Th1 chemokine CXCL10 plays an important role in the pathogenesis of GD, and that vitamin D analog inhibits CXCL10 in human thyroid cells [10, 11]. In addition, vitamin D analog inhibits inflammatory responses relating to autoimmune process of the development of GD [11, 12]. Although further study would be necessary to conclude, these and our present results suggest that low vitamin D values may influence the development and clinical outcome of GD.

In conclusion, serum vitamin D levels were significantly lower in female GD patients without remission than in those with remission. It is noted, however, this study is cross-sectional survey with a small number of subjects, and limited in its ability to conclude that vitamin D status is directly related to the pathogenesis and/or prognosis of GD. Therefore, the direct role of vitamin D in patients with GD should be examined by further prospective clinical studies by the treatment of vitamin D and experimental studies.
